# Elevated CD39+T-Regulatory Cells and Reduced Levels of Adenosine Indicate a Role for Tolerogenic Signals in the Progression from Moderate to Severe COVID-19

**DOI:** 10.3390/ijms242417614

**Published:** 2023-12-18

**Authors:** Alaa Elsaghir, Ehsan M. W. El-Sabaa, Asmaa M. Zahran, Sahar A. Mandour, Eman H. Salama, Sahar Aboulfotuh, Reham M. El-Morshedy, Stefania Tocci, Ahmed Mohamed Mandour, Wael Esmat Ali, Lobna Abdel-Wahid, Ibrahim M. Sayed, Mohamed A. El-Mokhtar

**Affiliations:** 1 Department of Microbiology & Immunology, Faculty of Pharmacy, Assiut University, Assiut 71515, Egypt; 2 Department of Clinical Pathology, South Egypt Cancer Institute, Assiut University, Assiut 71515, Egypt; 3 Department of Microbiology and Immunology, Faculty of Pharmacy, Deraya University, Minia 11566, Egypt; 4 Department of Clinical Pathology, Faculty of Medicine, Sohag University, Sohag 82524, Egypt; 5 Department of Chest Diseases and Tuberculosis, Faculty of Medicine, Assiut University, Assiut 71515, Egypt; 6 Department of Biomedical & Nutritional Sciences, Zuckerberg College of Health Sciences, University of Massachusetts Lowell, Lowell, MA 01854, USA; 7 Department of Anesthesia and ICU, Faculty of Medicine, Assiut University, Assiut 71515, Egypt; 8 Department of Clinical Pathology, Faculty of Medicine, Al-Azhar University, Assiut Branch, Assiut 71524, Egypt; 9 Gastroenterology and Hepatology Unit, Internal Medicine Department, Assiut University, Assiut 71515, Egypt; 10 Gilbert & Rose-Marie Chagoury School of Medicine, Lebanese American University, Byblos P.O. Box 36, Lebanon; 11 Department of Medical Microbiology & Immunology, Faculty of Medicine, Assiut University, Assiut 71515, Egypt

**Keywords:** COVID-19, T-regulatory cells, ATP, CD39, CD73, adenosine

## Abstract

Viral infections trigger inflammation by controlling ATP release. CD39 ectoenzymes hydrolyze ATP/ADP to AMP, which is converted by CD73 into anti-inflammatory adenosine (ADO). ADO is an anti-inflammatory and immunosuppressant molecule which can enhance viral persistence and severity. The CD39-CD73-adenosine axis contributes to the immunosuppressive T-reg microenvironment and may affect COVID-19 disease progression. Here, we investigated the link between CD39 expression, mostly on T-regs, and levels of CD73, adenosine, and adenosine receptors with COVID-19 severity and progression. Our study included 73 hospitalized COVID-19 patients, of which 33 were moderately affected and 40 suffered from severe infection. A flow cytometric analysis was used to analyze the frequency of T-regulatory cells (T-regs), CD39+ T-regs, and CD39+CD4+ T-cells. Plasma concentrations of adenosine, IL-10, and TGF-β were quantified via an ELISA. An RT-qPCR was used to analyze the gene expression of CD73 and adenosine receptors (A1, A2A, A2B, and A3). T-reg cells were higher in COVID-19 patients compared to healthy controls (7.4 ± 0.79 vs. 2.4 ± 0.28; *p* < 0.0001). Patients also had a higher frequency of the CD39+ T-reg subset. In addition, patients who suffered from a severe form of the disease had higher CD39+ T-regs compared with moderately infected patients. CD39+CD4+ T cells were increased in patients compared to the control group. An analysis of serum adenosine levels showed a marked decrease in their levels in patients, particularly those suffering from severe illness. However, this was paralleled with a marked decline in the expression levels of CD73. IL-10 and TGF-β levels were higher in COVID-19; in addition, their values were also higher in the severe group. In conclusion, there are distinct immunological alterations in CD39+ lymphocyte subsets and a dysregulation in the adenosine signaling pathway in COVID-19 patients which may contribute to immune dysfunction and disease progression. Understanding these immunological alterations in the different immune cell subsets and adenosine signaling provides valuable insights into the pathogenesis of the disease and may contribute to the development of novel therapeutic approaches targeting specific immune mechanisms.

## 1. Introduction

COVID-19 is an inflammatory disorder induced by SARS Coronavirus 2 infection (SARS-CoV-2). The infection can occur without symptoms, or the symptoms could be mild or severe lower respiratory symptoms that might lead to breathing problems and death [1,2]. SARS-CoV-2 can trigger antiviral immunity and can also induce excessive inflammation in patients with a serious COVID-19 infection, resulting in several irregularities in the activity and function of immune cells [3].

T-regulatory cells (T-regs) are important modulators of host immunity [4], and they control autoimmunity [5]. They are a subtype of CD4-T cells commonly referred to as CD4+CD25+Foxp3+ T-regulatory cells [6]. T-regs control the immune system by hindering the expansion and stimulation of T-effector cells in inflamed areas by affecting cytokine release [7]. T-regs and Th17 cells suppress viral-specific T cells, causing persistent infection and disease development [8,9]. In addition, T-regs regulate the immune system through the release of inhibitory cytokines such as interleukin 10 (IL-10) and transforming growth factor-β (TGF-β) [10,11].

CD39 mediates the catabolism of energy molecules. The hydrolysis of adenosine triphosphates (ATP) and adenosine diphosphates (ADP) to adenosine monophosphates (AMP) is catalyzed by the ectoenzyme CD39. CD73 then changes the broken-down AMPs into anti-inflammatory adenosine (ADO), which interacts with adenosine receptors on T lymphocytes and antigen-presenting cells and mediates the immunosuppressive effects of these cells [12]. Therefore, CD39 is a functional T-reg marker [13]. CD4+CD25+CD39+ T-regs have stronger immunomodulatory properties than CD4+CD25+CD39- T-regs [14]. There is evidence linking CD39+ Tregs to viral infections such as HBV infection [15]. Increased numbers of CD39+ Tregs were reported in people with cancer and Human Immunodeficiency Virus (HIV), and a direct link was established between CD39 expression on Tregs and the emergence of tumors and the spread of AIDS [16,17,18].

There is evidence to suggest that changes in the expression levels and frequencies of CD39 and CD73 in leucocytic cells have an impact on the immunopathology of viral illnesses such as viral hepatitis, cytomegalovirus (CMV), and HIV [19,20,21,22]. Regarding SARS-CoV-2 infection, the levels of inflammatory cytokines and purinergic signaling pathways are altered in patients with COVID-19 [23]. There is also an indication that T-cell lymphocytes and monocytes exhibit altered ectoenzyme expression [24,25,26,27]. However, more studies are needed to determine whether changes to this pathway contribute to the excessive inflammatory responses and infection progression seen in COVID-19. In this study, we analyzed the frequency of CD39+ T-regs, CD73 expression profiles, plasma adenosine levels, and the discharge of immunosuppressive mediators such as IL-10 and TGF-β to clearly understand the significance of these molecules in the pathogenicity of SARS-CoV-2 infection and their link to disease severity.

## 2. Results

### 2.1. Sociodemographic and Clinical Presentations of the Study Populations

The age of COVID-19 patients ranged from 25 to 80 years (mean = 53.6 ± 1.6 years). Most of them were in the age range of 45–64 years. There was no difference between the patients and controls in terms of age or gender (Table 1).

Most patients had a dry cough (97.3%) and dyspnea (94.5%). About 86.3% had fever, more than two-thirds had sore throat, and about 63% of patients had expectoration, while diarrhea and headache were less common in this group (less than 10%). High blood pressure (60.3%), diabetes (43.8%), and ischemic heart disease (12.3%) were the furthermost predominant comorbidities.

### 2.2. The Frequency of T-Cell Phenotypes among COVID-19 Patients

**Figure 1:**
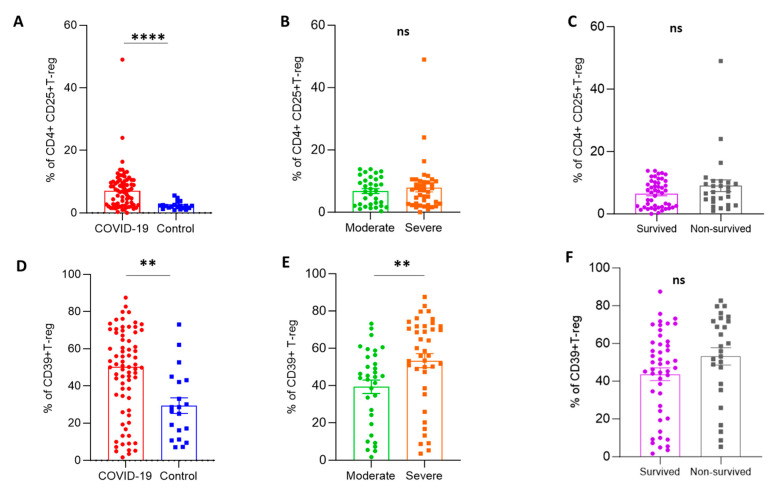
Frequency of different T-reg cells in the different studied groups (COVID-19; *n* = 73, moderate; *n* = 33, severe; *n* = 40, survived; *n* = 47, non-surviving; *n* = 26, and control; *n* = 20). (**A**–**C**) frequency of CD4+CD25+Foxp3+ T-cells (T-regs). (**D**–**F**) frequency of CD39+ T-reg. The Mann–Whitney test was applied to estimate the *p*-values between the various groups. Data were reported as means ± SEM. SEM: standard error of the mean, ns; non-significant; ** and **** indicate the *p*-values of <0.01 and 0.0001, respectively.

**Figure 2:**
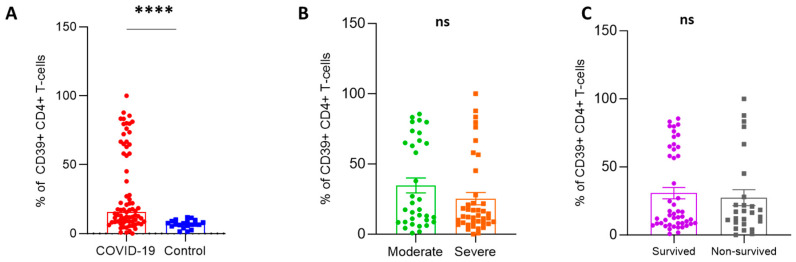
Frequency of CD39+CD4+ T-cells in COVID-19 patients with different outcomes and healthy controls. (**A**) The percentage of CD39+CD4+ T-cells was assessed in COVID-19 patients (*n* = 73) and healthy controls (*n* = 20). (**B**) The frequency of these cells was measured in moderate (*n* = 33), and severe (*n* = 40) COVID-19 patients. (**C**) The frequency of these cells was measured in surviving (*n* = 47) and non-surviving (*n* = 26) cases. **** mean *p*-value < 0.0001, as determined by a Mann–Whitney test. Data were reported as means ± SEM. ns; non-significant.

A flow cytometry technique was used to analyze the frequency of the following T-cell phenotypes among patient and control groups: CD4+CD25+Foxp3+ T-cells (T-regs), CD39+ T-reg, and CD39+CD4+ T-cells (Figure 1). The percentage of T-regs was significantly higher in patients than in controls (7.4 ± 0.79 vs. 2.4 ± 0.28; *p* < 0.0001), while there was no significant difference between severe and moderate cases and in disease outcome (Figure 1A,B). There was no difference in CD4+ T-reg cells between surviving and non-surviving cases (Figure 1C). Regarding CD39+ T-regs cells, COVID-19 patients had considerably greater frequencies of CD39+ T-regs compared to controls (47 ± 2.7 vs. 29 ± 4.2; *p* = 0.003). The proportion of CD39+ T-regs was elevated in severe cases compared to moderate cases (53 ± 3.8 vs. 39 ± 3.6; *p* = 0.003). Surviving patients were not significantly different from non-surviving cases (44 ± 3.4 vs. 53 ± 4.6; *p* = 0.06) (Figure 1D–F). Interestingly, there was a substantial increase in CD4+CD39+ T-cells in COVID-19 patients compared to controls (30 ± 3.4 vs. 6.8 ± 0.66; *p* < 0.0001). Patients with severe cases did not differ from those with moderate conditions (25 ± 4.4 vs. 35 ± 5.3; *p* = 0.23). Also, there was no difference between patients who survived and non-surviving individuals (31 ± 4.2 vs. 27 ± 5.8, *p* = 0.92), (Figure 2A–C).

### 2.3. Detection of Plasma Levels of Adenosine, IL-10, and TGF-β in Different Studied Groups

**Figure 3:**
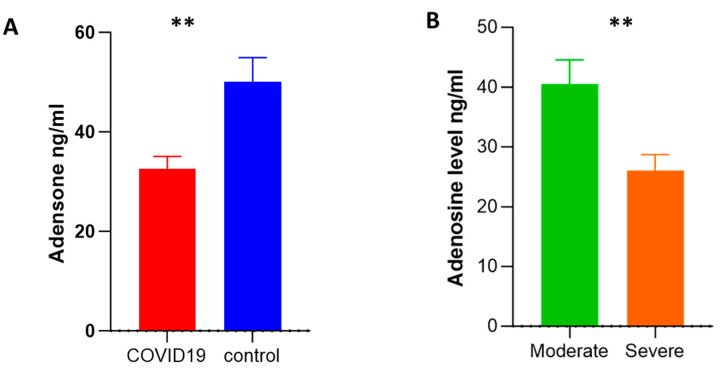
Assessment of the plasma level of adenosine via an ELISA. (**A**) The plasma level of adenosine (ng/mL) was compared in COVID-19 patients (*n* = 73) and healthy controls (*n* = 20). (**B**) The plasma level of adenosine was measured in moderate (*n* = 33) and severe (*n* = 40) COVID-19 patients. Data were reported as means ± SEM. ** *p*-value < 0.01, as determined by the Mann–Whitney test.

**Figure 4:**
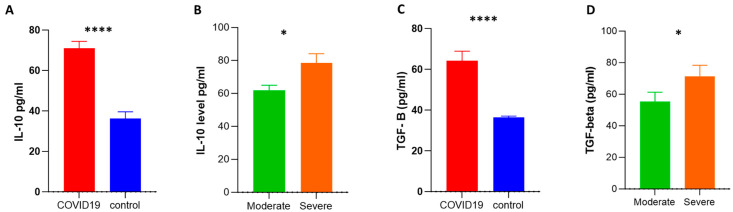
Assessment of plasma levels of anti-inflammatory cytokines via an ELISA. Plasma levels of IL-10 (pg/mL) (**A**,**B**) and TGF-β (pg/mL) (**C**,**D**) were measured by an ELISA. The Mann–Whitney test for non-parametric analysis was applied to estimate the *p*-value between the various groups. Data were reported as means ± SEM. SEM: standard error of the mean; * and **** indicate *p*-values of <0.05 and 0.0001, respectively.

We assessed the serum levels of adenosine and anti-inflammatory cytokines such as IL-10 and TGF-β. We selected the IL-10 and TGF-β cytokines since previous studies revealed that anti-inflammatory molecules such as IL-10 and TGF-β can be predictive indicators of medical severity, fibrosis of the lungs, and death in COVID-19 patients [28]. Adenosine levels in COVID-19 patients were significantly lower than in controls (32 ± 2.48 vs. 50 ± 4.8; respectively). Also, the level of adenosine was significantly lower in severe compared to moderate cases (26 ± 2.7 vs. 41 ± 4; respectively) (Figure 3A,B), while the levels of IL-10 and TGF-β were higher in COVID-19 patients than in healthy subjects, and the levels of these cytokines were significantly higher in severe cases than moderate cases (Figure 4A–D).

### 2.4. Assessment the mRNA Expression of CD73 and Adenosine Receptors in COVID-19 and Disease Outcome

**Figure 5:**
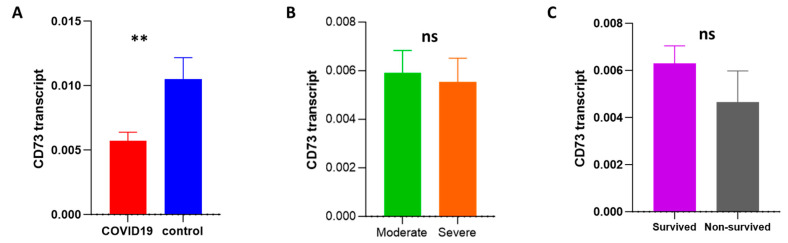
Assessment of the transcript level of CD73 among COVID-19 patients with different outcomes by an RT-qPCR. (**A**) The transcript level of CD73 was measured and compared among COVID-19 patients (*n* = 73) and controls (*n* = 20). The mRNA level of CD73 was compared between moderate (*n* = 33) and severe (*n* = 40) COVID-19 patients (**B**) and between surviving (*n* = 47) and non-surviving (*n* = 26) patients (**C**). ns: non-significant; ** *p*-value < 0.01, as determined by the Mann–Whitney test.

To investigate possible alterations in the mRNA expression of CD73 and adenosine receptors (A1, A2A, A2B, and A3) during infection, a gene expression analysis was carried out using an RT-qPCR. CD73 expression was significantly decreased in patients compared to the control group. However, the modulation of CD73 expression was not associated with alterations in disease progression or outcome (Figure 5A–C). Regarding adenosine receptor expression, there were no changes in the transcript levels of the adenosine receptors A1, A2A, A2B, and A3 (Supplementary Figures S1 and S2).

### 2.5. Logistic Regression Modeling for Predicting Possible Risk Variables for COVID-19 Severity

Risk predictors for infection severity were evaluated with a simple logistic regression approach for most variables. Table 2 shows the odds ratios (ORs) and 95% confidence intervals (CIs) for the studied parameters. We found that the neutrophil count, lymphocyte count, serum level of CRP, D-dimer and ferritin, SpO2%, and respiratory rate were significant risk variables for COVID-19 severity. Interestingly, we found that the percentage of CD39 (OR; 1.028, CI; 1.006–1.052; *p* = 0.01), IL-10 levels (OR; 1.0, CI; 1.005–1.044; *p* = 0.02), and adenosine concentration (OR; 0.96, CI; 0.93 to 0.99; *p* = 0.007) were strongly related to severity.

## 3. Discussion

T-reg cells are implicated as good cops and bad cops in disease progression and clinical outcomes. Although the main function of T-reg cells is to suppress the immune system through tolerance and immunoregulation, several reports showed that an imbalance in the level of T-reg cells could be linked to complications associated with COVID-19 infection [29,30]. Wang and colleagues showed that the percentage of T-reg cells was lower in COVID-19 patients than in controls, which could impact the inflammatory signaling pathways and the ratio of Treg/T-17 cells [29]. An imbalance in T-reg/T-17 cells could stimulate a cytokine storm [29]. Moreover, the neuropilin-1 receptor expressed on T-reg cells is linked with COVID-19-associated anosmia [31]. On the other hand, T-reg cells decrease susceptibility to SARS-CoV-2, increase tissue repair following infection, suppress hyperactivated immune cells, and can reduce some complications such as respiratory failure by affecting matrix metalloproteinases-9 protein [29]. While Galván-Peña et al. showed that greater amounts of T-reg cells and Foxp3 are associated with COVID-19 severity [30], T-reg cells could express a unique transcriptome signature and could possess antitumor and antiviral activities [30]. Therefore, the impact of T-reg cells on COVID-19 outcomes and patient prognosis is not completely understood. Interestingly, viral proteins such as the S-protein of the SARS-CoV-2 virus could affect T-reg development by affecting Foxp3 expression [32]. However, the previous finding has not been deeply studied.

The clinical symptoms and pathogenesis of SARS-CoV-2 infection are influenced by the host’s immune response. Excessive pro-inflammatory mediators have been observed in patients with severe COVID-19 [3]. CD39 and CD73 could mediate immune reactions through the degradation of ATP into adenosine [33,34]. Adenosine inhibits the body’s immunological response via its receptors, which may contribute to the severity of inflammatory diseases [35]. The aim of this study was to determine if CD39 expression, specifically on T-regs, correlates with the severity and development of COVID-19. We also determined whether CD73 and adenosine play a role in the immunopathology of SARS-CoV-2 infection and how that would affect the severity of the disease. Also, we examined the plasma levels of anti-inflammatory molecules such as adenosine, IL-10, and TGF-β as indicators of illness severity in patients.

In this study, the expression level of CD4+CD25+Foxp3+ T-regs was higher in COVID-19 patients than healthy controls. Ronit et al., who showed that T-reg cells were higher in the lungs than in blood during COVID-19 pathogenesis [36]. In addition, Galván-Peña et al. observed a considerable increase in Foxp3+ CD25^high^ cells in severely ill patients when compared to other cases [30]. However, other studies reported a significant decline in the frequency of T-regs in COVID-19 patients as well as a lower expression level of Foxp3 mRNA [37,38,39,40]. A possible explanation for why the levels of T-regs were elevated in our patients may be attributed to the enhanced positive anti-inflammatory response of T-regs, or it may be because, as concluded by De Biasi et al., that different groups of T-regs were elevated in blood circulation of COVID-19 with increasing the level of IL-10 cytokine [41].

Herein, we found that CD39+ T-regs was overexpressed in infected persons compared to healthy subjects and that this increase was more pronounced in severely ill patients than in those with moderate infections. Likewise, Simsek et al. identified a substantial increase in CD39+ T-regs in patients with mild-to-severe COVID-19 pneumonia [42]. Díaz-García et al. also reported that CD39 expression levels in CD4+CD25+Foxp3+ T-reg and CD14+monocytes cells were increased in patients relative to healthy controls [43]. Importantly, the expression of CD39 is upregulated in other viral illnesses such as hepatitis B virus infection [15], EBV [44], CMV [45], and HEV [46].

To assess the role of CD73 in the development of COVID-19 infection, we examined the transcript level of CD73 in the PBMCs of COVID-19 patients and matched controls. The gene expression of CD73 was considerably decreased in patients relative to healthy subjects. Consistent with our findings, Pietrobon et al. found that severe infections were related to reduced expression levels of multiple ectonucleotidase genes (ENPP1, ENPP2, ENPP3, and NT5E (CD73)) [47]. The expression of CD73 is reduced on cytotoxic lymphocytic cells such as CD8+ T, natural killer T (NKT) cells, and natural killer (NK) cells during COVID-19 [25]. Moreover, another study showed that there were decreased proportions of CD4+CD73+ and CD8+CD73+ in the circulating blood of severely infected COVID-19 individuals [26].

We then assessed the level of adenosine during COVID-19 infection. Adenosine is generated by the metabolization of extracellular ATP via the CD39/CD73 axis, and it has anti-inflammatory and immunosuppressant activities [48,49]. We observed a lower adenosine concentration in patients than in healthy individuals, as well as lower levels in severe cases compared to moderate cases. Similarly, Dorneles et al. found that people with COVID-19 had decreased adenosine concentrations in their circulation, which could lead to excessive inflammatory conditions and increase the severity of infection [26]. One possible explanation for the reduced adenosine concentration is decreased CD73 expression [26,47,50]. However, levels of IL-10 and TGF-β cytokines were higher in COVID-19 patients than in controls and were much higher in severe cases than moderate ones. Likewise, Pietrobon et al. showed that the ADO signaling pathway is disrupted in COVID-19 due to an increase the release of anti-inflammatory cytokines, especially IL-10 [47]. Similarly, a previous study found that people with severe COVID-19 had increased levels of IL-10 in their bodies compared to healthy people and people with mild COVID-19 [26]. The significant increase in IL-10 is a distinguishing hallmark of the cytokine storms occurring in COVID-19 [51]. Regarding TGF-β, several studies reported that COVID-19 patients had higher levels of TGF-β which were associated with the development of clinical symptoms such as cough and pneumonia [52,53,54].

Alterations in the expression of the adenosine receptors A1, A2A, A2B, and A3 have been documented to have direct effects on the inflammatory conditions in various diseases [55,56]. Consequently, we also studied adenosine receptor expression levels in the blood of infected participants and control subjects, and no discernible difference in adenosine receptor expression levels was observed. In a parallel line of thought, Pietrobon and colleagues reported that there was no substantial change in the genetic expression of any adenosine receptors except for A2A, which was considerably downregulated in blood samples from COVID-19 patients in critical condition [47]. These results suggested that COVID-19 infection may not directly alter the expression of adenosine receptors in the blood.

To investigate potential risk variables for the severity of COVID-19 infection, a simple logistic regression was made. Our results revealed that the higher percentage of CD39+T-regs, the increased secretion of IL-10, and the lower availability of adenosine are strongly related to the severity of the disease. These findings indicate the importance of CD39 and the associated signaling pathways in the pathogenesis of COVID-19 and determining the outcomes of infection.

In conclusion, we believe that enhanced CD39+ T cells play a role in COVID-19 pathogenesis. CD39, CD73 expression, adenosine, and associated cytokines contribute to the development of COVID-19. Adenosine deficiency may have a role in SARS-CoV-2 infection. Moreover, all these factors are also connected in some way to the progression of the disease. Understanding these immunological alterations in different immune cell subsets and adenosine signaling provides valuable insights into the pathogenesis of the disease and may contribute to the development of novel therapeutic approaches targeting specific immune mechanisms. However, this study did not include additional samples after recovery from infection, limiting the ability to provide insights into the post-recovery phase of SARS-CoV-2 infection.

## 4. Materials and Methods

### 4.1. Research Subjects and Ethics Considerations

This study was carried out in the period from November 2021 to May 2022 at the Department of Microbiology and Immunology, Assiut University, and the Department of Chest Diseases and Tuberculosis, Faculty of Medicine, Assiut University, in collaboration with the South Egypt Cancer Institute. This study’s proposal was accepted by the university’s review board (IRB: 17101485). It followed all guidelines set out in the Helsinki Declaration. Each eligible participant in the research gave informed written permission.

### 4.2. Study Subjects

This study included 73 individuals admitted to Assiut University Hospitals and confirmed to be infected with SARS-CoV-2 by an RT-qPCR and 20 healthy subjects of matched age and gender as controls. The patients’ demographic, clinical, and laboratory characteristics were provided. Depending on the severity of COVID-19 infection, patients were divided into moderate (*n* = 33) or severe (*n* = 40) groups, as defined by the American College of Emergency Physicians (ACEP) [57]. Cases were considered severe if they had any of the following: an oxygen saturation measured by pulse oximetry (SpO2) < 94% on room air at sea level, a ratio of arterial pressure of oxygen to the fraction of inspired oxygen (PaO2/FiO2) < 300 mm Hg, or a respiratory rate >30 breaths/min. Moderate cases were defined as those who exhibited evidence of lower respiratory illness upon clinical examination or CT scanning and whose oxygen saturation on room air at sea level was less than 94% on pulse oximetry (SpO2 ≥ 94%) [58]. The patients’ outcomes were recorded and based on the outcomes of the cases, one category included improved (survived) individuals (*n* = 47), and the other category included non-survived patients (*n* = 26).

Blood samples were collected and used for a flow cytometric analysis, a gene expression analysis, and cytokine assays.

### 4.3. Flow Cytometric (FCM) Analysis

**Figure 6:**
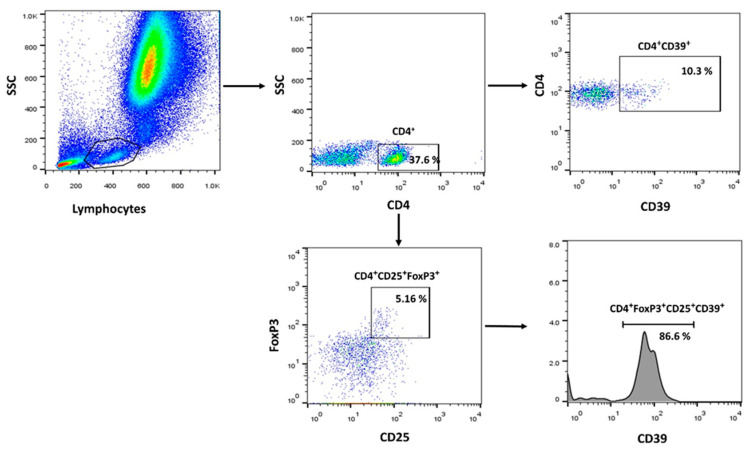
Gating strategy and illustrative histograms are used for identifying the distinct cell subsets.

The following antibodies were used to identify T-reg subsets by FCM: anti-hCD4 (PerCP-cy5.5, clone no: RPA-T4, Elabscience, Bio-Techne, Houston, TX, USA), anti-hCD25 (Alexa Fluor^®^ 488, clone no: 24212, R&D systems, Bio-Techne, MN, USA), anti-FoxP3 (APC, polyclonal Goat IgG, R&D systems, Bio-Techne, MN, USA), anti-hCD39 (PE, clone no: A1, Elabscience, Bio-Techne, Houston, TX, USA). Blood samples were first stained with the surface markers Alexa Fluor^®^ 488 anti-CD25, PerCP-cy5.5 anti-CD4, and PE-labeled anti-CD39. RBCs were then lysed using ACK lysis buffer. For intracellular FoxP3 staining, surface-stained cells were fixed and permeabilized using the FOXP3 Fix/Perm Buffer Set (BioLegend, San Diego, CA, USA), followed by staining with an anti-FoxP3 antibody. Following staining, the tubes were analyzed immediately. We included samples that were treated with isotype control antibodies to establish baseline fluorescence levels and ensure an accurate interpretation of specific antibody binding. DAPI dye (Invitrogen, Waltham, MA, USA) was used to identify dead cells. Dead cells and doublets were excluded from analysis. Data were acquired via a FACSCanto II flow cytometer (BD Biosciences, NJ, USA) and analyzed with FlowJo software 7.6.1 (Tree Star, Inc., Ashland, OR, USA). A representative gating strategy is shown in Figure 4. The frequencies of the following cell subtypes were analyzed: CD4+CD25+Foxp3+ T-cells (T-regs), CD4+CD25+Foxp3+CD39+ T-cells (CD39+ T-regs), and CD39+CD4+ T-cells. The gating strategy was presented in Figure 6.

### 4.4. Determination of Adenosine and Cytokine Profile by ELISA

The plasma levels of adenosine (Catalog No: EK700453, AFG Bioscience, Northbrook, IL, USA), IL-10 (Catalog No: E EL H0103, Elabscience, Bio-Techne, Houston, TX, USA), and TGF-β (Catalog No: E EL H0110, Elabscience, Bio-Techne, Houston, TX, USA) were estimated using an enzyme-linked immunosorbent assay (ELISA) according to the manufacturers’ instructions.

### 4.5. Quantitative Real Time PCR Analysis (RT-qPCR)

The expression levels of CD73 and adenosine receptors (A1, A2A, A2B, A3) were assessed using an RT-qPCR. cDNA was synthesized using the High-Capacity cDNA Reverse Transcription Kit (Applied Biosystems, Thermo Fisher Scientific, Waltham, MA, USA). The RT-qPCR was carried out using a WizPure™ qPCR Master (SYBR) (Wizbiosolutions, Gyeonggi-do, Republic of Korea), and the primers listed in Table 3. The expression GAPDH was used as a housekeeping gene for normalization. Results were expressed using the (2^−dCt^) method [59].

### 4.6. Statistical Analysis

Statistical tests were carried out using GraphPad Prism 8.3 (GraphPad Software Inc., SanDiego, CA, USA). Categorical variables are shown as numbers and percentages. Continuous measurements are shown as mean ± standard deviation (SD) or standard error of the mean (SEM) values as indicated. The Mann–Whitney test was used for comparisons between different groups. The severity risk factors were analyzed using a univariate logistic regression analysis. Differences were considered significant at *p* < 0.05.

## 5. Conclusions

In conclusion, we believe that enhanced CD39+ T cells play a role in COVID-19 pathogenesis. CD39, CD73 expression, adenosine, and associated cytokines contribute to the development of COVID-19. Adenosine deficiency may have a role in SARS-CoV-2 infection. Moreover, all these factors are also connected in some way to the progression of the disease. Understanding these immunological alterations in different immune cell subsets and adenosine signaling provides valuable insights into the pathogenesis of the disease and may contribute to the development of novel therapeutic approaches targeting specific immune mechanisms.

## Figures and Tables

**Table 1:** Age, gender, and clinical characteristics of patients and control group.

Variables		Patients, *n* = 73 (%)	Controls, *n* = 20 (%)	*p*-Value
Gender	Male	31 (42.5)	8 (40)	>0.99
	Female	42 (57.5)	12 (60)	
Age (years)	25–34 years	10 (13.7)	4 (20)	0.08
	35–44 years	7 (9.6)	3 (15)	
	45–54 years	18 (24.7)	7 (35)	
	55–64 years	21 (28.7)	4 (20)	
	≥65 years	17 (23.3)	2 (10)	
Clinical presentations	Cough	71 (97.3)	NA	NA
	Dyspnea	69 (94.5)	NA	
	Fever	63 (86.3)	NA	
	Sore throat	51 (69.9)	NA	
	Expectoration	46 (63)	NA	
	Anorexia	44 (60.3)	NA	
	Fatigue	37 (50.7)	NA	
	Myalgia	17 (23.3)	NA	
	Diarrhea	7 (9.6)	NA	
	Headache	2 (2.7)	NA	

NA: means not applicable. Healthy controls did not have the clinical presentations of the COVID-19 patients.

**Table 2:** Univariate logistic regression modeling for predicting probable risk variables for COVID-19 severity in the study population.

Variables	Univariate Logistic Regression (Severity)		
	Odds Ratio (OR)	95% Confidence Interval (95% CI)	*p*-Value
Age	1.00	0.98 to 1.0	0.46
Male	1.8	0.69 to 4.6	0.24
Leucocyte count	0.93	0.78 to 1.1	0.45
Neutrophils	1.1	1.0 to 1.2	0.008 *
Lymphocytes	0.79	0.7 to 0.88	<0.0001 *
CRP	1.0	1.0 to 1.1	0.04 *
D-dimer	1.5	1.1 to 2.2	0.02 *
Ferritin	1.002	1.001 to 1.003	0.01 *
Temperature	0.69	0.32 to 1.4	0.32
SpO2%	0.036	0.0016 to 0.18	0.003 *
Respiratory rate	1.9	1.5 to 2.6	<0.0001 *
CD39	1.028	1.006 to 1.052	0.01 *
CD73	0.93	0.47 to 1.8	0.81
Adenosine	0.96	0.93 to 0.99	0.007 *
IL-10	1.0	1.005 to 1.044	0.02 *
TGF-β	1.011	0.9987 to 1.025	0.08
Duration of hospital stay	1.1	0.96 to 1.1	0.53
Dyspnea	0.39	0.019 to 3.2	0.42

*: mean significant as *p* value is less than 0.05.

**Table 3:** Sequence of primers to be used in the qRT-PCR reactions.

Gene	Forward	Reverse	Ref.
GAPDH	GGAGCGAGATCCCTCCAAAAT	GGCTGTTGTCATACTTCTCATGG	[60]
CD73 (5′-nucleotidase ecto (NT5E)	GCCTGGGAGCTTACGATTTTG	TAGTGCCCTGGTACTGGTCG	[61]
adenosine A1 receptor (ADORA1)	CCTCCATCTCAGCTTTCCAG	AGTAGGTCTGTGGCCCAATG	[62]
adenosine A2a receptor (ADORA2A)	CTCCGGTACAATGGCTTGGT	TGGTTCTTGCCCTCCTTTGG	[63]
ADORA2B	ATGCCAACAGCTTGAATGGAT	GAGGTCACCTTCCTGGCAAC	
ADORA3	TTGACCAAAAGGAGGAGAAGT	AGTCACATCTGTTCAGTAGGAG	

## Data Availability

Data in this study are presented in the main text and Supplementary Materials. Please contact the corresponding author for further inquiries.

## Supplementary Materials

The supporting information can be downloaded at https://www.mdpi.com/article/10.3390/ijms242417614/s1.
