# Supplementary material for: Elevated CD39+T-Regulatory Cells and Reduced Levels of Adenosine Indicate a Role for Tolerogenic Signals in the Progression from Moderate to Severe COVID-19
Source: Int J Mol Sci. 2023 Dec 18;24(24):17614. doi: 10.3390/ijms242417614 (PMC10744088; doi:10.3390/ijms242417614)
Supplement: Supplementary file 1 [file ijms-24-17614-s001.zip › ijms-2744410-supplementary.pdf]

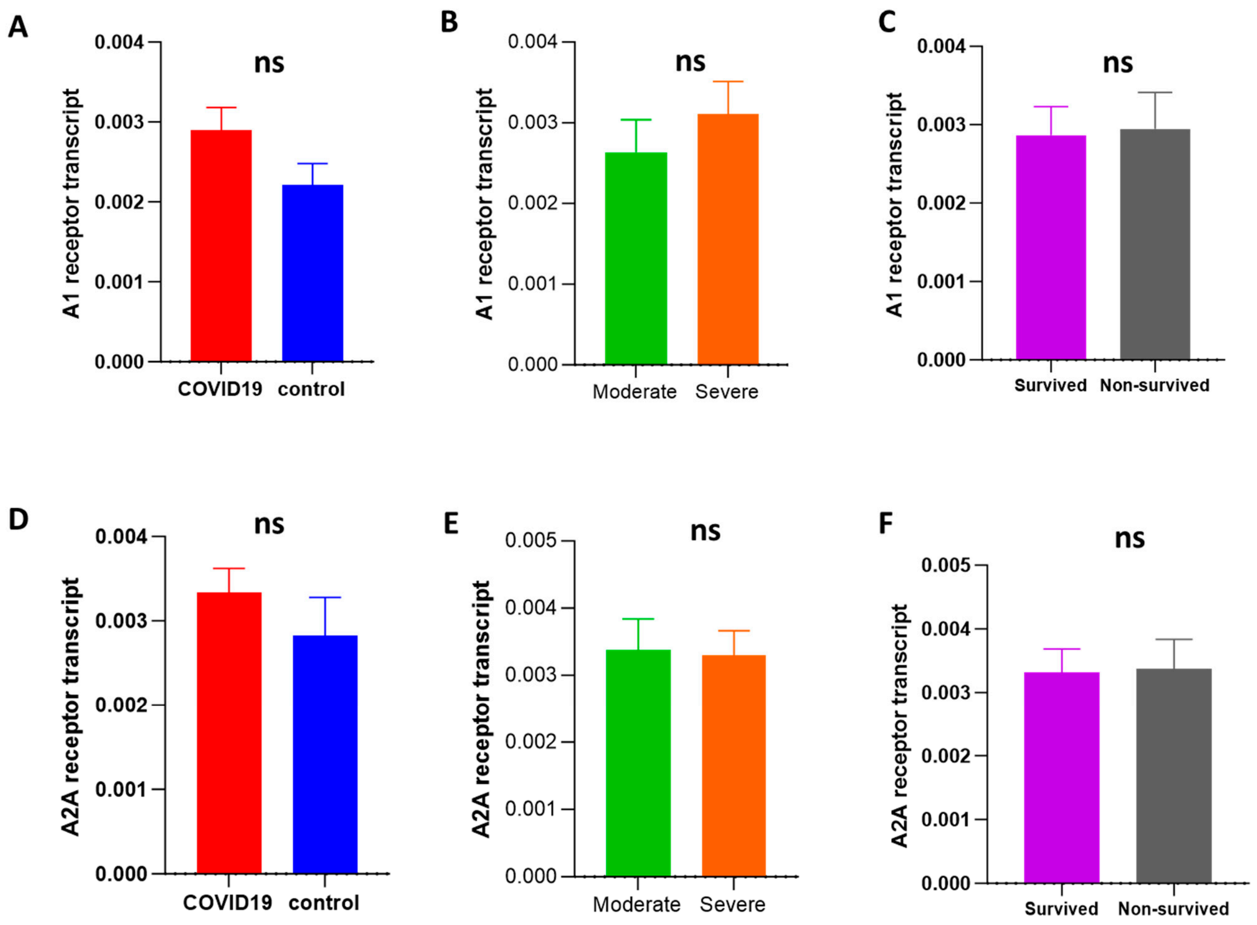

Supple Figure S1: Transcriptome expression of adenosine receptors A1 and A2A. (A, D) The transcript level of A1 and A2A receptors, respectively among COVID-19 and controls. (B, E) The transcript level of A1 and A2A receptors, respectively among severe COVID-19 and moderate COVID-19 patients. (C,F) The transcript level of A1 and A2A receptors, respectively among COVID-19 patients who survived and others are non-survived.

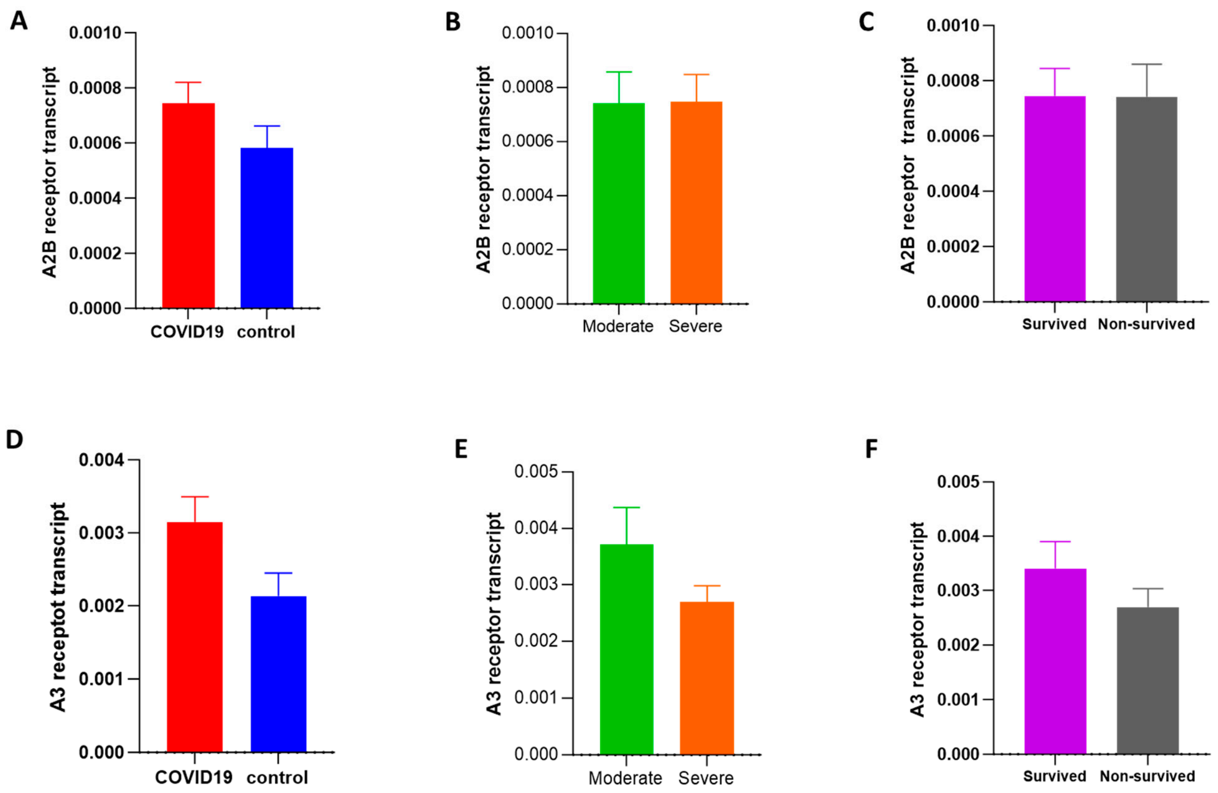

Supple Figure S2: Transcriptome expression of adenosine receptors A2B and A3. (A, D) The transcript level of A2B and A3 receptors, respectively among COVID-19 and controls. (B, E) The transcript level of A2B and A3 receptors, respectively among severe COVID-19 and moderate COVID-19 patients. (C,F) The transcript level of A2B and A3 receptors, respectively among COVID-19 patients who survived and others are non-survived.
